# Investigation of Polyurethane Matrix Membranes for Salivary Nitrate ISFETs to Prevent the Drift

**DOI:** 10.3390/s19122713

**Published:** 2019-06-17

**Authors:** Shuto Osaki, Takuya Kintoki, Takayo Moriuchi-Kawakami, Kenichi Kitamura, Shin-ichi Wakida

**Affiliations:** 1 Aist-Osaka University Advanced Photonics and Biosensing Open Innovation Laboratory, AIST, Suita 565-0043, Japan; osaki@ap.eng.osaka-u.ac.jp (S.O.); m1m18505@st.oit.ac.jp (T.K.); 2 Department of Applied Physics, Graduate School of Engineering, Osaka University, Suita 565-0043, Japan; 3 Department of Applied Chemistry, Graduate School of Engineering, Osaka Institute of Technology, Osaka 535-8585, Japan; takayo.moriuchi@oit.ac.jp; 4 National Institute of Technology, Toba College, Toba 517-8501, Japan; kitamura-k@toba-cmt.ac.jp

**Keywords:** ISFETs, ISE, polyurethane, salivary nitrate, stress, drift, ion-selective membrane

## Abstract

We have investigated human-stress monitoring by making use of salivary nitrate, which can be a candidate for stress markers, with ion-selective field-effect transistors (ISFETs). ISFETs are suitable for on-site single-drop analysis of salivary nitrate within 10 s. However, when ISFETs are used for salivary nitrate, ISFETs have a problem that is called the initial drift. The initial drift makes accurate nitrate monitoring difficult. Thus, the purpose of this study is to prevent the initial drift and to search for a new, simple polymer to possess a better performance of sensor responses than conventional matrix membranes, such as PVC. In this research, we investigated ISFETs using specific matrix membranes, for example KP-13, Pellethane^®­^_­_, and P7281-PU. The initial drift was evaluated from the fluctuations of the response values generated by the ISFETs when immersed in saliva or aqueous solution. As a result, P7281-PU showed a prevention effect on the initial drift, both in the whole saliva and in various solutions. Furthermore, the cause of drift may be H^+^ diffusion, and the drift prevention effect of P7281-PU may be affected by urethane bond capturing H^+^ in the ion-selective membrane. This result suggests that a continuous nitrate monitoring is feasible and may be applied to wearable sensors.

## 1. Introduction

Depression may be linked with stress and genetic vulnerability [1,2,3]. In addition, there are various diseases and physical disorders associated with stress. Therefore, stress measurement is an important issue and it contributes to the prevention of mental illness, such as depression. In this study, we are focusing on the biochemical method to evaluate stress from a stress-marker in body fluid.

One of the body fluids, blood, which contains typical stress-markers (adrenaline and noradrenaline [4,5]), is collected invasively accompanied by stress. In contrast, saliva, which is derived from blood [6], is collected non-invasively. Hence, saliva is suitable for stress-measurement. Salivary nitrate is secreted by blood nitrate, which is a Nitric Oxide (NO) metabolite, through the salivary grand [7]. NO is an endothelium-derived relaxing factor (EDRF) [8] that is produced in response to vascular tone associated with the effect of the autonomic nervous system. Therefore, salivary nitrate is expected to be a stress-marker of the autonomic nervous system. Other candidates for salivary stress-marker are cortisol, chromogranin A [9], and α-amylase [10]. However, these candidates are not suitable for on-site stress measurement because they are determined with enzyme-linked immune sorbent assay (ELISA), and it takes time to measure stress hormones. As measurement devices, ion-selective field-effect transistors (ISFETs) are possible for on-site single-drop analysis within 10 s. Hence, ISFETs are adapted for on-site stress measurements making use of saliva because the collected saliva is small volume, and degradation occurs immediately. On the other hand, current salivary NO_3_-ISFETs have the problem that drift occurs during measurement. Drift is the phenomenon in which the response value of the sensor changes gradually, making it difficult to determine salivary nitrate accurately. The ISEFTs’ gate part consists of a H^+^ sensitive metal oxide, for example, SiO_2_, S_3_N_4_, or Ta_2_O_5_. Fogt, et al. considered that the cause of the drift is the pH change at the ion-selective membrane (ISM)/gate interface’s aqueous layer. It is concerned with CO_2_-gas diffusion, which approaches the ISM/gate interface’s aqueous layer through the ion-selective membrane [11]. The gate part, which we use in this paper, is Ta_2_O_5_. Hereinafter, such pH-sensitive ISFETs are called pH-ISFETs. Other previous researches attempted to insert an inner layer in the ISM/gate interface, for example, pHEMA-gel saturated buffer [12], Ag/AgCl [13], and H^+^-selective membrane [14]. On the other hand, Abramova, et al. considered that the cause of drift is protein adsorption on the ISM surface. Photocurable polyurethane as a matrix membrane is effective in the measurement of blood plasma [15]. However, there is no report that aims to prevent drift in saliva. Therefore, we have investigated polyurethane as the matrix membrane to prevent drift for salivary nitrate. The actual used polyurethanes are Pellethane^®^, KP-13, and P7281-PU. Pellethane^®^ is widely used in the medical field, for example, as a catheter. Hence, Pellethane^®^ is expected to prevent the adsorption of glycoproteins in saliva. Espadastorre and Meyerhoff reported that Pellethane^®^-based ISM showed somewhat less platelet adhesion [16]. KP-13 is the polyurethane urea containing 13 wt % of dimethylsiloxane. Wakida reported that KP-13 is an effective matrix membrane to measure blood electrolytes [17] and salivary nitrate [18]. Although P7281-PU has not been used as a matrix membrane, P7281-PU used as an ISM has adhesiveness and can be closely adhered to the gate to restrict appearance of the water layer.

## 2. Materials and Methods

### 2.1. Materials

**Figure 1:**
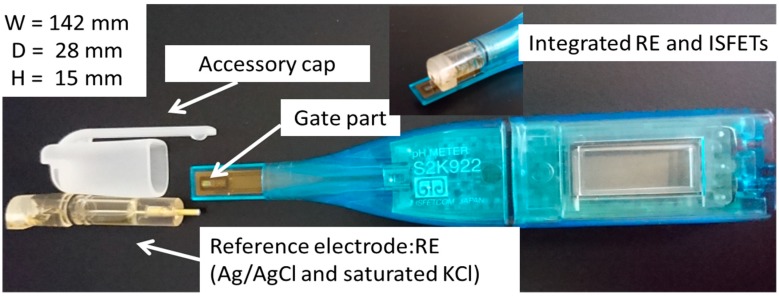
pH-sensitive ion-selective field-effect transistors (pH-ISFETs) and accessory cap.

Original ISFETs for pH measurement (pH-ISFETs) were purchased from ISFETCOM (Saitama, Japan). The pH-ISFETs have an integrated reference electrode (see Figure 1). NO_3_-ISFETs, which we used, are originated from pH sensor. This means that if the indicated value changes by 1.0, then the potential difference changes by about 59 mV. As a nitrate ionophore, Bis(dimethyl-phenanthroline) Copper(I) nitrate ([Cu(bcp)_2_]NO_3_) was synthesized as described in reference [19]. As a plasticizer, 2-Nitrophenyl dodecyl ether (NPDDE) was purchased from Wako Pure Chemical Industries (Osaka, Japan). NPDDE was used for stable ISFET to improve the adhesion to the gate material of the ISFETs [20]. Polyvinyl chloride (PVC, *n* = 1000), used as a matrix membrane, was purchased from Kishida Chemical (Osaka, Japan). KP-13 was received from Kaneka Chemistry (Osaka, Japan). Pellethane^®^ was received from Lubrizol (Wickliffe, OH, USA). P7281-PU was purchased from Polymer source (Dorval, QC, Canada).

NaCl, KCl, KH_2_PO_4_, urea, Na_2_SO_4_, NH_4_Cl, CaCl_2_·2H_2_O, KSCN, NaHCO_3_, NaNO_2_, KNO_3_, and tetrahydrofuran (THF) was purchased from Wako. THF was used as a volatile solvent to dissolve the nitrate ionophore, plasticizer, and polymer. Mucin from bovine submaxillary glands was purchased from MP Biomedicals, LLC (Santa Ana, CA, USA). Real saliva was collected by Salivette purchased from Sarstedt (North Rhine-Westphalia, Nümbrecht, Germany) and a metal spoon.

All chemicals were regent grade, except for NaNO_2_, and used without purification. NaNO_2_ was volumetric analysis grade. All standard solutions and artificial saliva were prepared with Milli-Q water (18.2 MΩ cm).

### 2.2. Preparation of NO_3_-ISFETs without Purification

Nitrate-selective ISFETs (NO_3_-ISFETs) were prepared by the following procedure. We dissolved completely and made the THF solution which forms the nitrate-selective membrane, containing 5 wt % of the nitrate ionophore ([Cu(bcp)_2_]NO_3_), 65 wt % of the plasticizer (NPDDE) and 30 wt % of the polymer matrixes. We cast the THF solution onto the gate part of the pH-ISFETs. After this casting, THF solution on the gate part was evaporated in a clean space (Pure Space 01; AS ONE, Osaka, Japan), and this was repeated several times. After this, the nitrate-selective membrane was completely dried for half a day. The thickness of the nitrate-selective membrane was ca. 0.2 mm. We estimate the value of 0.2 mm of the ion-selective membrane thickness from the amount of THF solution, which was filled in the gate part of ISFETs. In regard to the properties of the membrane surface, we consider that it is not out of order, because we paid attention to make the edge of deposited polymer layers out of the gate part of ISFETs and to make the membrane flat.

### 2.3. Determination of Calibration Curves and Selectivity Coefficients in NO_3_-ISFETs

After preparation, the ISFETs were conditioned in 1 mM (mol/L) KNO_3_ solution (standard solution) for 3 h. Calibration curves and selectivity coefficients were determined using the Nicolsky–Eisenman Equation (1), where *E* is the potential of the NO_3_-ISFETs, *E_0_* is the standard potential of NO_3_-ISFETs, *Zi* and *Zj* are the charges of the primary ion *i* (NO_3_) and interfering ions *j*, and $a_{i}$ and $a_{j}$ are the activities of ions *i* and *j*, respectively. *R*, *T*, and *F* have the usual meanings.

(1) $$E={E\;}_{0}+2.303\cdot \mathrm{RT}/\left(\mathrm{ZiF}\right)\cdot \ln\left(a_{i}+K_{ij}^{pot}a_{j}^{zi/zj}\right)$$

According to the theoretical response, at 25 °C, the slope sensitivity of the NO_3_-ISFETs is −59.16 mV per decade change of the activity *ai*. *ai* was calculated from the Debye–Huckel equation based on the simple ionic-strength theory. $K_{ij}^{pot}$ is the selectivity coefficient of the NO_3_-ISFETs in the presence of an interfering ion, *j*. $K_{ij}^{pot}$ was evaluated by the mixed-solution method and can be considered to be a reliable selectivity parameter. The potential responses were measured in NO_3_ standard solutions in the presence of each interfering ion using 5.0 × 10^−1^ M NaCl, 5.0 × 10^−1^ M Na_2_SO_4_, 5.0 × 10^−2^ M NaNO_2_, 5.0 × 10^−5^ KI, and 5.0 × 10^−5^ KSCN. Furthermore, the calibration curve in artificial saliva is also determined. Artificial saliva is an aqueous solution reproducing human salivary electrolytes. Reagents used for artificial saliva and their concentrations are as follows: 2.15 mM NaCl, 12.9 mM KCl, 4.81 mM KH_2_PO_4_, 0.33 mM Urea, 2.37 mM Na_2_SO_4_, 3.33 mM NH_4_Cl, 1.55 mM CaCl_2_·2H_2_O, 7.51 mM NaHCO_3_.

### 2.4. Evaluation of Initial Drift of the NO_3_-ISFETs

**Figure A1:**
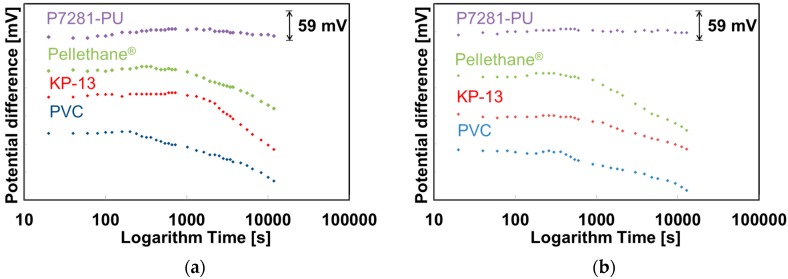
Initial drift of NO_3_-ISFETs in whole saliva. (**a**) Same data as Figure 3f; (**b**) for reproducibility check.

Evaluation of the initial drift was performed by looking at the output value of the ISFETs immersed in solution for about 8 h. The main measurement target was human saliva. Two types of human saliva samples were prepared. One was obtained from a saliva collection kit, Salivette^®^, and the other was obtained from a metal spoon. Four kinds of solutions, 10^−3^ M potassium nitrate (standard solution), artificial saliva, the standard solution with 0.5 wt % Mucin (mucin solution), and standard solution with CO_2_ bubbling (CO_2_ solution), were used for comparison. For the artificial saliva used for this section, 1 mM KNO_3_ was added. In the experiments, evaluation of the initial drift was conducted in a closed system using a cap attached to the ISFETs in order to prevent volatilization of the aqueous solution and contact with air (see Figure 1). We changed an ISM every measurement. Evaluation of drift was carried out several times for one kind of sample. Regarding device reproducibility, we refer to the Appendix A (see Figure A1). These results show the same fluctuation of value, which is not completely corresponded. We think the drift of these fluctuation trends is repeated at any time. On the other hand, these fluctuations do not correspond to the results because of ISFETs’ characteristics. This point of non-correspondence is the focus of future work.

All subjects gave their informed consent for inclusion before they participated in the study. The study was conducted in accordance with the Declaration of Helsinki, and the saliva sample experiments were approved by the Ethics Committee of National Institute of Advanced Industrial Science and Technology (AIST) (Project identification code is human 2015-195) on 29 January 2016.

## 3. Results and Discussion

### 3.1. Calibration Curves and Selectivity Coefficients

**Figure 2:**
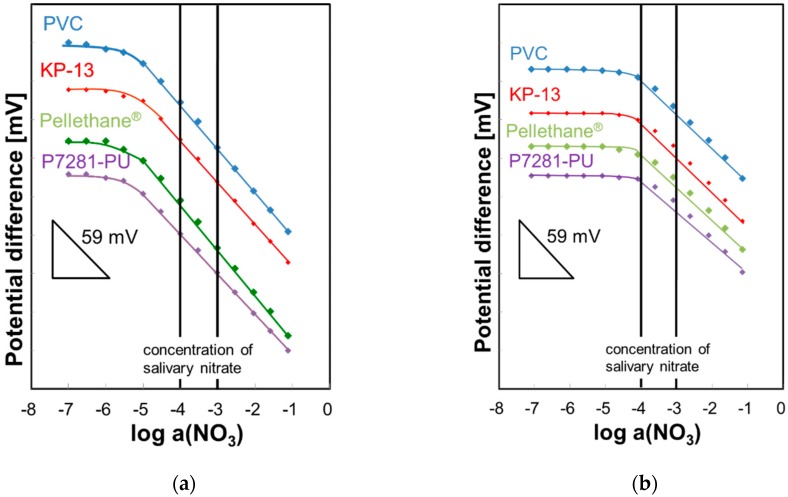
Calibration curves of NO_3_-ISFETs. (**a**) Only KNO_3_ solution. (**b**) In artificial saliva.

The calibration curves of the NO_3_-ISFETs are shown in Figure 2a. The *y* axis shows the potential difference and the *x* axis shows the logarithm of the nitrate activity. The calibration curves are arranged by the starting points so that they are easier to understand. Therefore, we insert the indicator of the slope sensitivity in Figure 2. All NO_3_-ISFETs show a linear response to nitrate activity in the range 10^−5^ M to 10^−1^ M. The selectivity of the ISFETs is represented by the selectivity coefficients $K_{NO_{3}}^{pot}$ defined by the Nicolsky–Eisenman equation. Selectivity coefficients for typical anions were measured by the mixed solution method and are summarized in Table 1. We obtained acceptable selectivity for salivary nitrate determination. The linear response range and the selectivity coefficients of the polyurethane-based ISFETs coincide with those of the PVC-based ISFETs. Therefore, potential determinants are ionophore and plasticizer but not matrix membrane. The calibration curves in artificial saliva are shown in Figure 2b. All ISFETs show a linear response to nitrate activity in the range of 10^−4^ M to 10^−1^ M. The concentration range of nitrate in saliva is approximately 10^−4^ M to 10^−3^ M [21]. Therefore, the NO_3_-ISFETs are adapted to nitrate measurement in saliva.

### 3.2. Evaluation of Initial Drift in Saliva and Various Solutions

**Figure 3:**
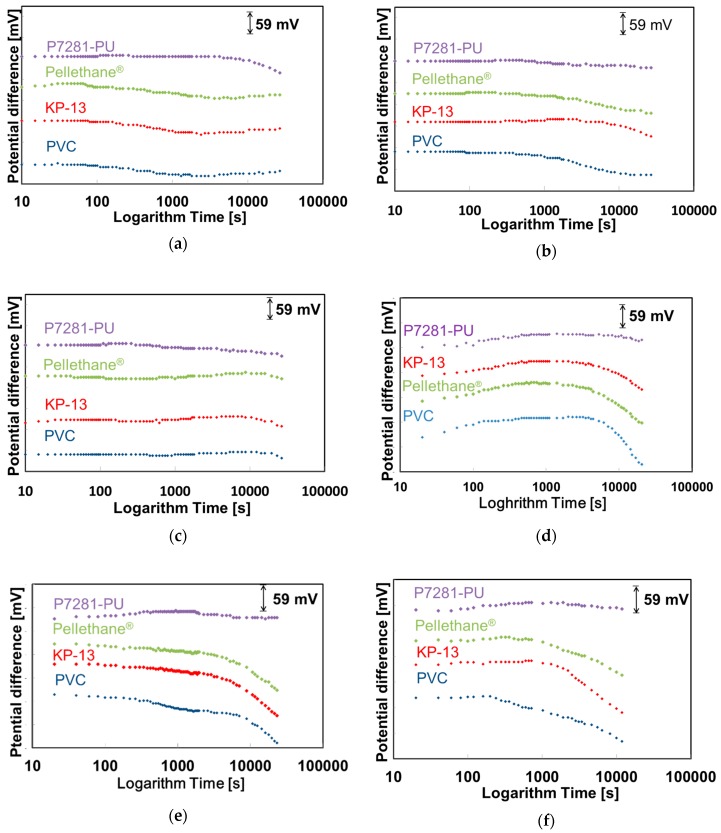
Initial drift of NO_3_-ISFETs (**a**) in standard solution; (**b**) in standard solution with 0.5 wt % mucin; (**c**) in standard solution with CO_2_ bubbling; (**d**) in artificial saliva; (**e**) in saliva via Salivette; (**f**) in whole saliva (non-pretreatment).

**Figure 4:**
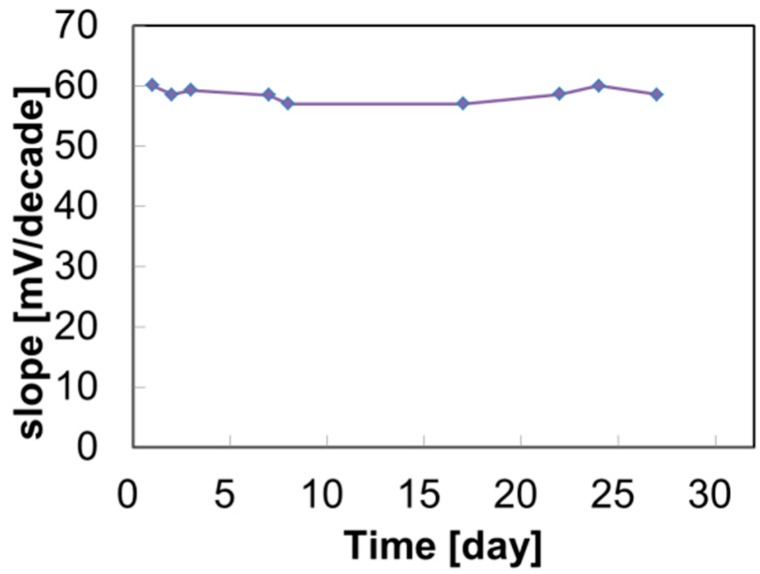
Long-term stability of P7281-PU-based ISFETs. This measurement is done in only KNO_3_ solution.

The drift characteristics in saliva and various solutions are shown in Figure 3. The *y* axis shows the potential difference and the *x* axis shows the logarithm of time. The results of drift are arranged by the starting points so that they are easier to understand. Therefore, we insert the indicator of the potential difference in Figure 2. In standard solution and CO_2_ solution, all NO_3_-ISFETs show a comparatively stable response (see Figure 3a,c). In mucin solution, the PVC-based ISFETs show drifts slightly, but the polyurethane-based ISFETs show a comparatively stable response (see Figure 3c). The results of Figure 3c suggest that CO_2_ is not the cause of drift. Furthermore, mucin is the cause of drift for PVC-based ISFETs but less so for the polyurethane-based ISFETs. PVC, KP-13, and Pellethane^®^-based ISFETs show drift in salivary samples (see Figure 3d–f). However, P7281-PU-based ISFETs show a comparatively stable response in salivary samples. Figure 4 shows the long-term stability of P7281-PU-based ISFETs. The *y* axis shows the slope sensitivity and the *x* axis shows the time. P7281-PU-based ISFETs maintain the same slope sensitivity for at least 27 days. It is considered that the stable response of the P7281-PU-based ISFETs is due to the strong adhesion of the ion-selective membrane to the Ta_2_O_5_ gate and that the formation of water layer at the ISM/gate interface is prevented.

**Figure 5:**
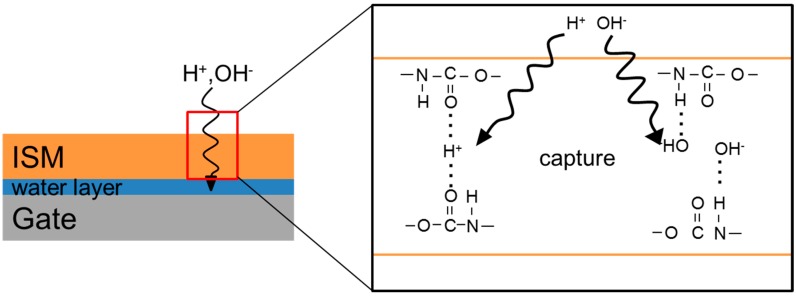
The hypothesis of urethane bond capture of H^+^ and OH^-^ by hydrogen bonds (in whole saliva).

Note that the drift characteristics of the P7281-PU-based ISFETs are extremely stable in whole saliva. It is considered that urethane bond in P7281-PU captures H^+^, OH^-^ in the aqueous layer. This hypothesis is suggested in Figure 5.

However, the cause of drift is not clear, and it is currently being investigated using matrix membranes that have the same molecular structure as P7281-PU but only differ in molecular weight. In any case, P7281-PU is an effective matrix membrane for NO_3_-ISFETs for measurement of saliva. In the future, it can be expected to achieve continuous nitrate monitoring and deployment to wearable sensors. In addition, we could conduct further experiments in different pH or temperature solutions to elucidate the cause of the drift.

## Figures and Tables

**Table 1:** Selectivity coefficients.

Polymer Matrix	$\mathrm{Selectivity}\;\mathrm{Coefficients},\;K_{NO_{3}^{-},j}^{pot}$				
	I	SCN	NO_2_	Cl	SO_4_
PVC	0.82	0.95	−1.53	−2.55	−4.10
KP-13	0.56	0.95	−1.53	−2.59	−4.03
Pellethane^®^	0.78	1.09	−1.50	−2.71	−4.11
P7281-PU	0.69	1.07	−1.40	−2.30	−4.13

## Appendix A

To investigate the reproducibility of NO_3_-ISFETs, we show the two drift results in Figure A1. The sample for this test is whole saliva, and the method is the same as in the main text (Section 2.4, Evaluation of Initial Drift of the NO_3_-ISFETs).
